# Developing Digital Media to Destigmatize Emergency Department Human Immunodeficiency Virus Testing Among Sexual and Racial Minority Youth: A Hyper-iterative Methodology

**DOI:** 10.7759/cureus.7209

**Published:** 2020-03-08

**Authors:** Ian D Aronson, Robert Freeman, Tonya Taylor, Alex S Bennett

**Affiliations:** 1 Research, Digital Health Empowerment, Brooklyn, USA; 2 Health Communication, New York University School of Global Public Health, New York, USA; 3 Medicine: Infectious Disease, State University of New York (SUNY) Downstate Medical Center, Brooklyn, USA; 4 Research, New York University School of Global Public Health, New York, USA

**Keywords:** hiv testing, youth, video, technology, emergency department, stigma

## Abstract

Rates of undiagnosed youth human immunodeficiency virus (HIV) remain problematically high across the United States and internationally. In addition, youth HIV test rates remain consistently low, and youth with HIV remain undiagnosed for longer periods of time as compared with older populations. Youth HIV remains especially persistent among African American adolescents and emerging adults, who are less likely to have consistent access to primary care and thus to HIV testing and prevention education. Therefore, increasing youth HIV test rates has become an important priority in emergency departments and other settings. At the same time, many young patients may not disclose risk behaviors or even engage in discussions of HIV testing when they interact with healthcare providers because they may fear being stigmatized.

Technology-based interventions present valuable opportunities to reframe risk reporting and discussions of testing by designing computer-mediated interactions that young sexual and racial minority participants find non-judgmental and less threatening. If designed in accordance with empirically tested theories of instructional design/multimedia learning and established models of behavior change, technology-based interventions can increase the number of HIV tests offered to young people and offer testing in nonthreatening ways that more young people will accept. The current paper describes a hyper-iterative methodology used to develop the Mobile Augmented Screening (MAS) tool, a technology-based intervention designed to destigmatize HIV and increase HIV test rates among youth.

## Introduction

In the United States, youth with HIV are the least likely (as compared to other age groups) to receive timely linkage to care or have a suppressed viral load [1]. Almost three-quarters of sexually active high school students have not tested for HIV and more than half of young people with HIV remain undiagnosed [2-3]. A survey of sexually experienced gay and bisexual males aged 14-18 years indicates that despite increased risk as a population, less than one-third had ever tested for HIV and nearly half did not know where they could test [4].

In addition, sexual and racial minority youth continue to face multiple stigmas that can result in medical mistreatment, which, in turn, might contribute to youths’ decisions to decline HIV testing [5-6]. Prior research indicates young people, and especially African American youth, frequently will not disclose risk factors when interacting with health care providers because they fear being stigmatized simply for discussing HIV [7].

Further, many young people who do test may not understand the need to re-test if they have been recently exposed to HIV or if they continue to engage in behaviors that increase their risk of HIV infection [8]. To address these issues, our team sought to develop a nonthreatening video-based intervention that would encourage young emergency department (ED) patients to report HIV risk and test for HIV.

Our team’s previous research has shown that brief computer-based video interventions (<15 minutes) can increase HIV test rates in EDs, including HIV testing among young patients aged 18-24 years who initially decline HIV testing offered by hospital staff [9-10]. As we developed the current intervention, we hypothesized that we could improve effectiveness by optimizing the content for a population of adolescents and young adults. Given the considerable barriers to testing and prevention that arise from stigma, we also chose to focus on using media to destigmatize not only the HIV test process but HIV itself.

The Information, Motivation, Behavioral skills model (IMB) and Social Cognitive Theory (SCT) both posit that intervention materials should be made relevant to specific populations; in this case, youth aged 13-24 years[11-14]. For a message to result in behavior change, according to SCT, an intervention recipient must first decide the message is relevant and worth attending to [13]. Questions, therefore, emerged as to how we could create content about HIV testing, prevention, and care that would appear relevant to young viewers who often believe themselves not at risk and who are unlikely to test for HIV if they do not show symptoms [15].

Rather than create multiple versions of the complete intervention and run several different effectiveness trials, which would have been prohibitively expensive and time-consuming, we conducted multiple rounds of formative research before beginning production to determine what content a diverse group of young people thought would most effectively increase HIV testing, re-testing, and facilitate linkage to care as needed. Specifically, we employed a highly iterative series of formative and summative evaluations to examine participant response to intervention elements, and then refined our content based on multiple waves of feedback from members of our target population [16]. The current paper describes both our methodology and our findings.

## Materials and methods

Methods

Participants

We recruited a convenience sample of adolescents and young adults aged 13-24 (n=43) in a high-volume New York City ED that serves the upper Manhattan area, including Harlem, which has one of the City’s highest concentrations of HIV. As described in the following sections, we worked with this sample to develop, refine, and evaluate a brief video designed to encourage youth HIV testing and re-testing.

Among these 43 participants, 42% were female and 58% were male. Forty-four percent of participants identified as Black or African American, with 28% identifying as Black non-Hispanic and 16% as Black Hispanic. Thirty-five percent of participants identified as White, with 12% identifying as White non-Hispanic and 23% identifying as White Hispanic. Nine percent of participants identified as multiracial, and less than 1% of participants identified as Asian. Twenty-three percent of participants were between the ages of 13 and 16, 35% were between the ages of 17 and 20, and 42% were between the ages of 21 and 24. The mean age of all participants was 19.3; all were recruited in 13 days of data collection in Autumn 2016 and Spring 2017.

Procedure

Video Production and Evaluation

To develop a video that addressed stigma and was specifically targeted to a young population, we collected consecutive waves of qualitative data to more deeply examine themes that emerged during each round. As explained below, this approach was built upon iterative methodologies adopted from the field of instructional design (ID) [16]. A key ID process entails eliciting feedback from potential users of educational materials at multiple points (before, during, and after production) to help optimize effectiveness. Thus, we first conducted a set of formative interviews with young ED patients (n=19) followed by two levels of evaluation: a formative evaluation, in which additional young patients (n=12) responded to a set of storyboards (paper representations of draft video content); and a summative evaluation in which a third group of young ED patients (n=12) reviewed an intervention video we created in response to participant feedback elicited during earlier rounds of interviews. Our goal was to seek repeated rounds of input and refine our design to the greatest extent possible, before creating our final product.

Research Assistants (RAs) recruited participants who were not known to be HIV positive, not intoxicated, and understood written and spoken English. All participants provided written informed consent if aged 18 or older; younger patients provided assent and written parental consent. The Principal Investigator (PI) developed an interview guide as well as a target enrollment table to ensure the project sample was representative of the larger hospital population in terms of race/ethnicity and gender and included a broad age range of patients between the ages of 13 and 24 years. All consent forms, instruments, and interview guides were approved by governing Institutional Review Boards.

RAs approached patients in the ED after they had initially been seen by a physician and were waiting for the next step in their treatment (e.g., an X-ray or blood work). All interviews were audio-recorded. Participants were given $25 gift cards to compensate them for their time.

After obtaining written consent, RAs conducted brief (<20 minute) semi-structured interviews. For the formative interviews, participants were asked who they would like to see appear in a video designed to encourage youth aged 13-24 to agree to test for HIV and why, and what possible scenarios might most encourage youth to test. For instance, during the first phase of interviews participants were asked questions, such as “Would you pay more attention to the video of someone your own age or someone older?” and “Do you think a video for young people would be effective if we show a doctor or is there someone else you would want to see in the video?”

During the second phase, the formative evaluation, RAs approached a new set of ED patients. Participants evaluated and compared two multi-panel storyboards, each of which used stock images or still images from our team’s prior videos to depict potential video scenarios informed by findings from the first round of interviews. The first storyboard consisted of three images: a young female on a swing saying she was diagnosed with HIV when she was 16 years old, an oral HIV test kit with details about the testing procedure in a word bubble (e.g. the test takes 20 minutes and does not require a blood draw), and a health care provider (HCP) discussing HIV rates among youth. The second storyboard consisted of four images: a young woman stating that she tested because her friends had tested; a young woman jogging accompanied by the caption HIV “doesn't stop me from living my life”; a still image from one of our team’s earlier videos depicting an HCP talking to a patient about youth HIV rates; and a still image from the earlier video of the same patient taking an oral HIV test, along with text noting that the test only takes 20 minutes and stating that treatment is available for those who test positive. Among the questions asked of participants in the formative evaluation phase were, “Are the people you see in the storyboard the appropriate people or is there someone else that should appear?” and “Would it encourage you to test for HIV if you saw this in a video?”

Participants in the summative evaluation completed a four-item HIV knowledge pre-test, watched the brief (<6 minute) video that our team developed based on the aforementioned systematic analysis of responses elicited during the initial formative interviews and the formative evaluation interviews, then completed a four-item HIV knowledge post-test and an automated set of acceptability items. Knowledge test items were selected based on HIV-related knowledge deficits identified among youth in previous research, and our team has successfully used different versions of these questions in previous NIH-funded studies [8-10]. Acceptability items, which our team has also used successfully in previous studies, consisted of a series of questions and participants were asked to mark their response on an accompanying scale from 1 to 10. For example, the question “How much did you understand the program you just completed?” appeared above a line labeled with “Not at all” on the left end of the line at 1 and “Very much” at the right end of the line at 10.

The video depicted three HIV-positive, non-actors describing how they became infected with HIV, their history living with HIV, the risks associated with HIV, the importance of testing, and the benefits of pre- and post-exposure prophylaxis (PrEP and PEP). One of the people depicted was an African-American female, one was a Hispanic male, and the third was a mixed-race female. Each appeared onscreen and spoke into the camera as if they were directly addressing the viewer. They spoke about very personal aspects of their experience, for example, one woman described being in a monogamous relationship and contracting HIV from her partner who had not known he was HIV positive.

In addition, an African-American member of the research team appeared in the video dressed as a medical professional (she wore a white lab coat and draped a stethoscope around her neck) who provided HIV-related statistics and described the rapid oral HIV testing procedure, which was demonstrated onscreen. RAs then conducted a brief (<20 minute) interview in which participants were asked questions regarding what they remembered most about the video, how the video could be improved, and whether the video encouraged or discouraged HIV testing.

## Results

Formative interviews

Responding to questions regarding who should appear in a video, youth participants in the formative interviews frequently cited relatability as the characteristic that would most likely motivate them to test for HIV. Specifically, participants said (1) they would be more likely to relate to, and, therefore, accept information from individuals who are age-appropriate (e.g., appear to be aged 13-24) and not professional actors, (2) that personal narratives and statistical information should be presented by youth and physicians, respectively, and (3) that the video should be realistic about HIV-related risks without unnecessarily alarming the viewer.

The majority of participants were insistent that, although the gender and race/ethnicity of those who were to appear in the video was relatively unimportant, stating, for instance, that “I think HIV doesn’t discriminate” (21-year-old White, non-Hispanic female), it was nonetheless important that information be presented by individuals close to participants’ age range. Moreover, most participants (16 out of 19) differentiated between information provided by individuals living with HIV, who one participant described as “real people actually going through [life with HIV] and not actors” (17-year-old Black, Hispanic female) and information delivered by a medical professional. Participants noted they would prefer informative personal narratives to come from HIV-positive peers and factual information such as testing details and HIV statistics to come from a physician.

The doctor will try to tell you what to do in order to keep yourself healthy and people with the actual experience would talk about how personally it affects their personal lives and stuff and how they feel walking around.

Twenty-four-year-old African American, non-Hispanic female

Themes of de-stigmatization immediately began to emerge. For example, participants stressed that although the video’s message should be somewhat cautionary (i.e. accurately warning of the risks of HIV), the video should not show negative images of HIV positive youth, but, instead, should be mostly uplifting and positive (14-year-old Black, Hispanic Male). As a 20-year-old White, non-Hispanic female suggested, the video should be, “like something that’s inspirational or like makes you feel better or something.”

Formative Evaluation

Participants in the formative evaluation also noted they preferred a younger cast, more relatable language, and hearing cautionary tales from individuals living with HIV in a positive, life-affirming manner. For instance, when asked what should be included in a video to best capture young people’s attention, one participant responded as follows: “Their life stories. That would be interesting and [to] know how they got [HIV] and how they feel or what they are going through and how their body is changing from having it and the treatments” (24-year-old Black, non-Hispanic female). During this phase of interviews, as described earlier, participants were shown two storyboards, which both drew heavily on suggestions made during the initial interviews.

Comparing a 16-year-old female who appeared in one storyboard looking at the ground, to a young woman in a second storyboard who was jogging and saying HIV “doesn't stop me from living my life,” participants suggested they found the first to look “sad” and “depressed” (17-year-old Hispanic female), and noted that this type of negative depiction of HIV positive youth would most likely not encourage young people to test, stating, for instance, that “The girl in the swing [looks] really sad. I always see that in every commercial. I’m just like, oh gross,” (24-year-old Asian female) and that, “She just looks really sad and that probably discourages kids” (17-year-old Hispanic female). Meanwhile, all of the participants who viewed the storyboards favorably viewed the young woman jogging, saying they approved of the message that if one were to test positive for HIV, it would not mean that they could no longer live a positive, fulfilling life.

Similar to participants in the formative interviews, participants who viewed the storyboards suggested that personal narratives from HIV positive individuals living healthy lives, alongside factual statistical information from a medical professional, would capture the attention of young ED viewers and would encourage them to test.

Because these people actually like are - they have HIV so they can touch the hearts but the doctors wouldn’t. [The doctors] could just be like you know, this is HIV, this is that but the kids are just going to be like that’s not enough evidence. Now the people that actually do have HIV is going to back up the evidence and tell them like you know, like I messed up, use protection always, things like that so they can encourage the kids more.

Thirteen-year-old Black Hispanic male

Summative interviews

During the summative interviews, consistent with previous analyses, every participant who watched the video responded favorably to receiving non-stigmatizing inspirational messages from an HIV-positive peer. For instance, after viewing the final video, one young woman noted that the positive nature of the testimony in the video encouraged her to test because a positive diagnosis would not mean life was over.

I remember one particular girl; she said that your life doesn't automatically stop, if you get HIV. You can still live a normal life, but there are some cautions you should take to prevent from getting it. [...] Because me, personally, when I think of HIV, I’m like, ‘Oh my God, it's the end of the world,’ but they seemed to be pretty healthy, and carrying on with her daily lives.

Seventeen-year-old Hispanic female

Participants also explicitly noted that the video effectively conveyed the HIV-related risks faced even by people in trusting, monogamous relationships. One young woman onscreen described her decision to stop using condoms with her long-term boyfriend and then becoming infected because the boyfriend had not known he was HIV positive. Participants described this as highly effective, as evidenced in the response of a 21-year-old Black, Hispanic female who said, “Yeah, it encouraged me to test for sure like you know she was unaware, she didn't know.”

Effects of Video on Youth Knowledge of HIV

Nine out of 12 (75%) summative evaluation participants did not know at baseline that HIV testing can be done without drawing blood, five (41.7%) did not know HIV test results can be available in 20 minutes, and five (41.7%) did not know some people who test negative for HIV may need to re-test in 90 days.

Eight people (66.7 %) did not know people can increase their risk of HIV infection if they “drink alcohol or smoke weed before sex.” After watching the video, which explained that using alcohol or other drugs can hinder a person’s attentiveness or judgment thus putting them at increased risk of HIV, some participants (3 out of 12) objected to the wording of the question, saying they thought it implied a direct causal relationship between substance use and HIV. Nonetheless, as one participant noted, such a message might still be effective if it remains realistic:

So you can tell them to be careful while drinking alcohol, and be aware of their surroundings and their actions, and be cautious of their alcohol intake, but not to say “Don't consume alcohol,” because people will. [...] Because they're giving you a different message of avoiding HIV - from you contacting HIV.

Seventeen-year-old Hispanic female

Additionally, several participants reported they were surprised to learn that people infected with HIV do not necessarily show symptoms. Interviews revealed a commonly held assumption that feeling healthy is a strong indication that there is no need to test for HIV, as illustrated by the following response from a participant who watched the video:

It was that like they said even if you don't feel like you're not healthy or like you feel healthy like you just still get tested because you never know. [That stood out] because it shows that like you can still like be able to feel better and stuff, but still maybe something wrong with you that you don't know.

Fourteen-year-old, Black, non-Hispanic female

After viewing the final video, significant increases were found for all four knowledge items. Also, after watching the video all but one participant answered all four items correctly (please see Table 1).

**Table 1:** Knowledge test results, pre-post (N=12) HIV: human immunodeficiency virus

Question	Percent Correct Pre	Percent Correct Post	Significance
Under some circumstances, a person who tests negative for HIV may need to re-test in 90 days.	58.3	91.7	p=.04
People who drink alcohol before sex or who smoke weed before sex increase their risk of HIV infection.	33.3	91.7	p=.002
HIV test results can be available in 20 minutes.	58.3	91.7	p=.04
HIV testing can be done without drawing blood.	25	91.6	p=.001

Eleven of the 12 participants interviewed as part of the Phase III summative evaluation indicated that upon learning new information about HIV testing from the video, they were either re-evaluating their decision to decline an HIV test or felt a desire to re-test if they had tested in the recent past (the one who did not, indicated that she didn’t need to test because she was not sexually active).

Sometimes I don't [test when offered] and now y’all made me feel like I should. Like today I said no, but that's because I got tested in December, so I felt like I [didn’t] need to, but then now, I'm kind of like I should, because 90 days are coming up.

Twenty-one-year-old, Black, Hispanic female

Video Acceptability

As described earlier, each item consisted of a single question, and participants were asked to mark their responses on an accompanying scale from 1 to 10. Mean acceptability scores for each item were very high, with the exception of one item measuring how threatening participants found the video, which was very low (indicating participants did not find the intervention threatening).

Mean scores for each item appear in Table 2, along with standard deviations.

**Table 2:** Acceptability scores, mean and standard deviation, per item

Acceptability Item	Mean Score	SD
How interesting was the program you just completed?	7.81	2.51
How useful was the program you just completed?	7.97	1.91
How much new information did you learn as a result of the program you just completed?	7.44	2.18
How easy to use was the section of the program you just completed?	7.88	1.33
How much did you understand the program you just completed?	8.51	1.06
How much did you like the program you just completed?	6.84	2.10
How threatening did you find the program you just completed?	3.92	3.29

## Discussion

Our highly iterative design enabled us to develop a more thorough and nuanced understanding of what types of intervention content could encourage youth HIV testing and organically address issues of stigma. Key themes began to emerge in our first set of video development interviews, and we were able to explore them more deeply in subsequent rounds. Participants overwhelmingly favored images and messaging that portrayed individuals who tested positive for HIV as feeling healthy and optimistic about life. For instance, participants expressed particular appreciation for a healthy-looking woman who discloses her HIV positive status in the video and tells viewers that “Being diagnosed with HIV doesn’t mean that you’re a bad person or that you did something wrong. You can be careful and still become infected with HIV.”

The finding that participants consistently said the gender and race/ethnicity of people appearing in the video were less important compared to their perceived authenticity aligns with previous findings by our team and others. For example, Jemmott et al. found that an HIV risk reduction intervention originally designed for teenage African American females was not less effective among teenage Latina females [17]. Similarly, prior research by our team indicates intervention videos designed to increase HIV testing among adult ED patients were not less effective when the people onscreen were not demographically concordant with the viewer in terms of race and gender compared to when the people onscreen matched the gender and/or race of the participant [18].

Interestingly, these findings also align with IMB and SCT that, as described above, both prescribe making interventions relevant to target populations [11-14]. Although intervention developers frequently attempt to make materials relevant via demographic concordance, our current findings suggest that the credibility of non-actors describing their own lived experience, compared to someone who lacks personal experience and is reading from a script, may be enough to establish the relevance required for people to attend to the message of behavior change [18]. Additional research with a larger sample is warranted, and our team is currently conducting a clinical trial to further examine the effectiveness of our intervention materials in an exceptionally high volume pediatric ED.

In the meantime, the knowledge tests and acceptability measures administered during the summative evaluation suggest our approach is promising. The pre-test results indicate fundamental, and potentially dangerous, knowledge gaps persist among youth. In fact, our results show that participants continue to lack foundational knowledge of HIV, including knowledge deficits identified in previous research by Swenson et al. in 2010 [8]. Post-test results showing that our five-minute video can potentially address key issues of HIV knowledge are very encouraging and warrant further research. The high acceptability scores indicate our target audience finds the video nonthreatening and easy to understand - two key measures for a population that not only lacks HIV knowledge but historically fears stigma related to testing and discussions of prevention.

The most important result of the current study may ultimately be the development of our hyper-iterative methodology to create more effective digital media by integrating theories of instructional design and multimedia learning. In contrast to what Mayer (2005) would call a technology-centered approach, in which developers ask how they can maximize capabilities of the newest multimedia technologies to create better interventions, we employed a human-centered approach that focuses on adapting technology to fit the cognitive functions of the intervention recipient [19]. In our case, this meant conducting successive waves of interviews and evaluations to determine what content would be most effective in an intervention video; who should deliver the content; and what tone the content should take, then refining our materials in response to each round of feedback. By describing our methodology here, along with our initial results, we hope to provide a model that can inform future technology-based interventions to not only destigmatize HIV and further increase testing but that can address other sensitive behavioral health issues as well.

## Conclusions

In conclusion, by depicting young people living with HIV as maintaining healthy, fulfilling lives, this tablet-based intervention has the potential to destigmatize HIV testing among hard-to-reach populations of young ED patients. Additionally, emphasizing the fact that a positive HIV diagnosis is not an indication that an individual has “done something wrong” and that anyone can potentially become infected with the virus may help to destigmatize being HIV positive in general. Further, our methodology of eliciting suggestions from sexual and racial minority youth regarding the form and content of a video designed to encourage HIV prevention, testing, and treatment facilitates the inclusion of diverse perspectives that might otherwise not be represented in a behavioral health intervention.
